# Phenotypic plasticity in mass loss during chick rearing in the European starling (*Sturnus vulgaris*)

**DOI:** 10.1002/ece3.11028

**Published:** 2024-02-23

**Authors:** Brett L. Hodinka, Tony D. Williams

**Affiliations:** ^1^ Department of Biological Sciences Simon Fraser University Burnaby British Columbia Canada

**Keywords:** birds, European starling, mass loss, parental care, phenotypic plasticity, reproduction

## Abstract

It has long been recognized that mass loss during breeding could be adaptive (e.g., by ameliorating the costs of increased parental activity). However, many studies still commonly interpret mass loss as evidence of “stress” or a cost of reproduction (i.e., a negative effect of high workload during chick provisioning). Despite several studies reporting evidence in support of both hypotheses, the ecological and/or life‐history contexts under which mass loss may be viewed as a “cost” or an adaptive strategy are still unclear. Here, we used a long‐term dataset from a breeding population of European starlings (*Sturnus vulgaris*) to investigate natural annual and individual variation in body mass and mass loss and to test whether mass loss during chick rearing represents a phenotypically plastic trait that varies predictably in relation to ecological context and individual quality. While there was significant annual variation in incubation mass, chick‐rearing mass, and mass change, there were no systematic relationships between mass loss and current breeding success or future fecundity and survival. In addition, we found no evidence of intra‐annual repeatability of mass loss between first and second broods ( = .00) but moderate interannual repeatability of mass loss (*R* = .61) during first broods, suggesting differences in mass loss under different selective pressures. However, we found no covariation between residual intra‐individual variation in mass loss for first broods and other reproductive or life‐history traits. We therefore found no support for the idea that mass loss reflects “reproductive stress” in our system: there were no negative relationships between mass loss and either current or future reproduction and survival (local return rate). Our results are consistent with mass loss being an individually plastic trait, with individuals using mass loss to “level the playing field” and individually optimize reproductive effort and fitness within their specific ecological context and relative to their individual quality for a given breeding attempt.

## Graphical abstract

It has long been recognized that avian mass loss during breeding could be adaptive, though many studies still commonly interpret mass loss as evidence of “stress” or a cost of reproduction. Here, we used a long‐term dataset from a breeding population of European starlings to investigate the ecological and/or life‐history contexts under which mass loss may be viewed as a “cost” or an adaptive strategy and, specifically, test whether mass loss during chick rearing represents a phenotypically plastic trait that varies predictably in relation to ecological context and individual quality. Overall, we found no support for the idea that mass loss reflects “reproductive stress” in our system and instead suggest that mass loss may represent an individually plastic trait that individuals use to “level the playing field” and individually optimize reproductive effort and fitness.

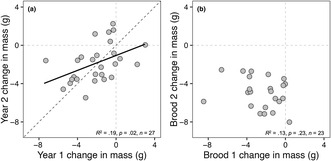

## 1 INTRODUCTION

During the breeding season, females of many altricial avian species gain mass prior to egg laying, maintain mass during incubation, and then lose mass during chick rearing (reviewed in Moreno, 1989; Swanson, 2010). It is often assumed that parental mass loss during chick rearing reflects “stress” associated with sustained, high‐intensity activity (sensu Drent & Daan, 1980) required for provisioning chicks (the *reproductive stress hypothesis*; e.g., Askenmo, 1977; Hussell, 1972; reviewed in Ricklefs, 1974; Swanson, 2010). Consequently, mass loss is often used as an index of reproductive costs (Bryant, 1988; De Steven, 1980; Nur, 1984). However, an alternative view, also long recognized, is that mass loss could be adaptive, allowing birds to ameliorate the costs of increased activity during parental care (the flight‐adaptation or adaptive mass loss *hypothesis*; reviewed in Moreno, 1989; but see Blem & Blem, 2006; Cichoń, 2001; Freed, 1981). Since flight is costly (9.2× basal metabolic rate; Butler, 2016) and this cost is mass‐dependent (Freed, 1981; Goldstein, 1988; Walsberg, 1983), abrupt, or stepwise loss of mass at, or preceding, the peak energetic demands of feeding nestlings should reduce the amount of energy required for parental care and increase parental fitness (Croll et al., 1991; Norberg, 1981). In addition, reduced mass might decrease risk of predation (Gosler, 1996; Gosler et al., 1995; Witter & Cuthill, 1993) during the chick‐rearing stage.

Although many studies have investigated mass change during reproduction in birds, most of these have concluded that empirical data either support one or the other hypothesis (reviewed in Swanson, 2010); that is, these represent binary outcomes. However, Moreno (1989) acknowledged that patterns of mass change during reproduction might not be simple consequences of reproductive stress but the outcome of “adaptive compromises between different selective factors.” Similarly, Ritz (2007) highlighted that the two mass loss hypotheses might not be mutually exclusive and that, while mass loss in females could be stress‐induced, the amount of mass lost might still be an adaptive adjustment, in this case related to the reliability of the food supply. Indeed, Hillström (1995) proposed the benefit–cost hypothesis, which predicts that parents should decrease their body mass to a level that optimizes parental investment and maximizes life‐time reproductive success. In this context, few studies have (a) compared patterns of mass change across multiple years where selective factors might indeed be different and (b) determined whether mass loss varies within as well as between individuals in different ecological contexts (i.e., whether it is a repeatable or individually plastic trait). Instead, studies to date have collected data over the course of just one or 2 years (e.g., Boyle et al., 2012; Croll et al., 1991; Jones, 1994; Ritz, 2007) and have not differentiated “good” from “bad” years in their study system beyond providing speculative relationships between body mass and annual prey availability (e.g., Jones, 1994; Ritz, 2007, but see Wendeln & Becker, 1996). Additionally, Potti and Merino (1997) reported low, nonsignificant, interannual repeatability of mass loss between the incubation and brooding stages in female pied flycatchers (*Ficedula hypoleuca*; *R* = .23) and when environmental variables were accounted for, repeatability was even lower (*R* = −.10). They concluded that “female mass loss apparently lacks additive genetic variance” in their population. Furthermore, to our knowledge no study has investigated long‐term fitness consequences of female mass loss in terms of future fecundity (e.g., during second broods or in subsequent years) and survival, to test Hillström's (1995) idea that individual variation in mass loss maximizes life‐time reproductive success.

In this study, we used long‐term data (2012–2022) from a free‐living population of European starlings (*Sturnus vulgaris*) to determine if mass loss during chick rearing represents a phenotypically plastic trait that varies predictably in relation to ecological context and individual quality. Our specific objectives were to determine (a) if there was annual variation in mass loss, if this variation was associated with annual variation in incubation mass, chick‐rearing mass or both, and if this differed between first and second broods (since other selective factors differ markedly between breeding attempts in our system; Cornell & Williams, 2016); (b) if individual variation in mass loss is explained by early reproductive decisions (lay date, clutch size, and incubation period), measures of workload (provisioning rate and mate contribution), and current breeding success (brood size and chick quality); and (c) if individual variation in mass loss is predictive of future fecundity (in second broods and the subsequent year) and survival (local return rate). We predicted that, if mass loss reflected reproductive stress then there should be consistent negative relationships between mass loss, current reproduction, and, in particular, future fecundity and survival (a “cost of reproduction”). Similarly, for reproductive stress, we predicted low or no repeatability of mass loss because individuals would likely experience different levels of “stress” in subsequent breeding attempts in different ecological contexts.

## 2 MATERIALS AND METHODS

Fieldwork was conducted at the Davistead Farm in Langley, British Columbia, Canada (49°10′ N, 122°50′ W) between April and July 2012–2022 using a free‐living population of European starlings. The field site is comprised of approximately 150 nest boxes mounted on fence posts around open pastures and on farm structures. In each year, a constant basic field protocol was followed: nest boxes were checked daily from April 1 to determine lay date, clutch size, hatch day, and brood size at hatch; day 6 post‐hatch; and fledging (day 21 post‐hatch). Morphometric measures including mass, wing chord, and tarsus length were collected from adult females at day 6 incubation (6.3 ± 1.3 days, *n* = 567) and day 10 chick‐rearing (9.6 ± 1.3 day, *n* = 279). Mass loss was quantified for all individuals captured during both incubation and chick‐rearing stages (*n* = 252). Here, “mass loss” is defined as the difference between day 6 incubation mass and day 10 chick‐rearing mass measures (approximately 15 days apart) of adult female starlings where incubation day is defined as the number of days after clutch completion and chick‐rearing day refers to the number of days post‐hatch. For reference, European starlings in our study system typically incubate for 11 days until hatch; chicks then remain in the nest for 21 days before fledging. Using tripod‐mounted video cameras (Canon VIXIA HF R800), 30–60 min behavioral observations of chick‐rearing parents were conducted during first broods at individual nest boxes from 9:00 to 15:00 h when chicks were 6–10 days old (2013–2022; *n* = 866 observations). Male and female provisioning rates (number of visits/30 min) were averaged across recording days for each nest box in each year (*n* = 315). From these data, change in mass data were available for 270 observations. Chick quality data are calculated as the mean mass of all chicks within a brood (*n* = 387 broods) and were collected on day 17 post‐hatch. Upon capture, adult females were sexed based on bill color (Feare, 1984) and fitted with individually numbered metal bands (Environment Canada Permit # 10646). After the fledging of first broods, data collection was repeated for second broods. In some years, we conducted experiments that potentially affected natural mass change of both adults and nestlings via wing‐clipping and/or attachment of radio‐transmitters (e.g., Allen et al., 2023; Fowler & Williams, 2017; Serota & Williams, 2019), so in this paper we excluded these and restricted analysis to nonmanipulated birds and controls. All protocols were approved by the Simon Fraser University Animal Care Committee (permit # 1172B‐96).

### 2.1 Statistical analysis

All statistical analyses were performed in RStudio v. 4.0.3 (R Core Team, 2023; RStudio Team, 2020). Residuals from all subsequent models were first examined for normality by visual examination of histograms and normal probability plots followed by the Shapiro‐Wilk test while homogeneity of variance tests were conducted using the Levene's test. Appropriate data transformations were conducted where necessary; but, in most cases models provided satisfactory distribution of residuals.

Linear mixed‐effects models (hereafter LMMs; R package “lme4”; Bates et al., 2015) were used to investigate annual variation in incubation mass, chick‐rearing mass, and change in mass (dependent variables) for first and second broods separately, with year as the main effect, tarsus as a covariate (to account for differences in body size), and bird ID as a random effect. For each model, we conducted interannual pairwise comparisons (R package “emmeans”; Lenth, 2020) with Bonferroni corrections to account for multiple comparisons. Unpaired *t*‐tests were used to test significance of change in mass (relative to zero) for each year and paired *t*‐tests with Bonferroni corrections were performed on the change in mass in each year for first and second broods, separately. Unpaired *t*‐tests with Bonferroni corrections were performed on interbrood comparisons of incubation mass, chick‐rearing mass, and change in mass within years and paired *t*‐tests were performed on a restricted dataset including individuals where change in mass data were available for both first and second broods within years. LMMs were also used to investigate individual variation in chick‐rearing mass and mass loss (dependent variables) with incubation mass as the main effect, tarsus as a covariate, and bird ID and year as random effects.

Three LMMs were used to investigate interannual variation in first brood incubation mass, chick‐rearing mass, and change in mass with year as a main effect, tarsus as a covariate, and bird ID as a random effect in each model. Similarly, three LMMs were used to investigate intra‐annual variation between first and second brood incubation mass, chick‐rearing mass, and change in mass with brood as a main effect, tarsus as a covariate and bird ID and year as random effects in each model. We then used the “rptR” package (Stoffel et al., 2017) to test interannual (year 1 vs. year 2) and intra‐annual (brood 1 vs. brood 2) repeatability of incubation mass, chick‐rearing mass, and change in mass; repeatability is a measure of within‐individual consistency in a trait over time, expressed as the proportion of total phenotypic variation accounted for by among‐individual variation (Lessells & Boag, 1987). Next, we used LMMs to explore the remaining proportion of variance from the interannual repeatability analysis (residual change in mass) that could be attributed to within‐individual differences across years in relation to changes in incubation mass, early reproductive decisions and annual mean‐corrected measures of current breeding success parameters (i.e., lay date, clutch size, incubation period, brood size at fledge), and workload (i.e., provisioning rate). All models included tarsus as a covariate and bird ID and year as random effects. Annual mean‐corrected measures of current breeding success were calculated by subtracting the population mean values from each individual's recorded value for that measure.

To explore individual variation in mass loss relative to current breeding success parameters and measures of workload, LMMs were used to (1) test the relationship between change in mass and lay date, brood size at day 6 and 21 post‐hatch and mean brood mass, and female provisioning rate and total provisioning rate and (2) investigate variation in mass loss relative to lay date, incubation period, and degree of male provisioning contribution where male provisioning rate was binned into four groups (i.e., visits/30 min; >6.00 = high, 3.00–5.99 = medium, 0.01–2.99 = low, 0.00 = none). All models included tarsus as a covariate and bird ID and year as random effects. We performed pairwise comparisons with Bonferroni corrections on mass loss by lay date, incubation period, and degree of male provisioning contribution.

To investigate individual variation in mass loss in relation to future fecundity and survival, we first calculated the total number of individuals that (1) attempted a second brood in the same breeding year, (2) successfully fledged at least one chick during a second brood attempt, and (3) returned in a subsequent year and attempted a first and/or second brood (local return rate). We use local return rate as a survival index given adult European starling survival rates are high among passerines (Cabe, 2020) and, in our study system, we have an approximately 95% success rate at identifying all nest box breeding females. We acknowledge that, while deferred breeding is uncommon among passerines, there may have been some banded individuals that bred in natural cavities. We then used LMMs to test the relationship between change in mass and each aforementioned dependent variable. Two LMMs were used to test the relationship between brood size at day 21 post‐hatch and total fecundity in a subsequent year (dependent variables) and change in mass (fixed effect) with tarsus as a covariate and bird ID and year as random effects.

## 3 RESULTS

### 3.1 Annual variation in incubation mass, chick‐rearing mass, and change in mass during reproduction

For first broods, change in body mass between incubation and chick rearing varied significantly among years (*F*_7,131_ = 3.15, *p* = .004, *n* = 177). Mass loss was overall significant when pooling years (−2.0 ± 2.4 g; *t*_176_ = 10.83, *p* < .0001; Table 1) with pairwise comparisons revealing significant mass loss in seven of 9 years (2012, 2016, and 2018–2022; *p* < .05 in all cases) but not in 2013–2014 (Table 1). Mass change was greatest in 2022 (−3.2 ± 2.6 g, *n* = 15) and smallest in 2014, with a slight gain between stages (0.7 ± 2.1 g, *n* = 10). There were relatively few significant pairwise differences among years, although mass gain in 2014 was significantly different from four other years with mass loss (2016, 2019, 2021, 2022; *p* < .05 in all cases). There was also significant annual variation in incubation mass (*F*_7,154_ = 5.28, *p* < .0001) and chick‐rearing mass (*F*_7,70_ = 3.26, *p* = .005) for first broods. However, there were more pairwise differences among years in incubation mass (six of nine) than chick‐rearing mass (one of nine), suggesting incubation mass was more variable.

**TABLE 1:** Annual variation in incubation mass, chick‐rearing mass, and change in mass for first and second broods of adult female European starlings.

Year	Brood	Incubation mass	*n*	Chick‐rearing mass	*n*	Change in mass	*n*	*t*	*p*
Pooled	1	81.5 ± 3.7	331	79.9 ± 3.4	187	−2.0 ± 2.4	177	10.83	<.0001
	2	82.8 ± 4.4	236	78.3 ± 3.4	92	−5.4 ± 2.8	75	16.47	<.0001
2012	1	81.2 ± 3.8	31	81.2 ± 2.7	19	−1.6 ± 2.5	14	2.34	.04
2012	2	83.2 ± 5.4	33	80.3 ± 4.0	10	−4.6 ± 4.1	9	3.30	.01
2013	1	80.8 ± 3.9	40	79.4 ± 2.9	18	−1.2 ± 2.6	18	2.01	.06
2014	1	81.3 ± 3.6	15	81.9 ± 3.9	10	0.7 ± 2.1	10	−1.11	.29
2016	1	82.9 ± 3.3	18	80.4 ± 3.6	12	−2.5 ± 1.6	12	5.30	<.001
2016	2	85.5 ± 2.6	7	–	–	–	–	–	–
2018	1	81.0 ± 4.1	54	79.2 ± 3.8	36	−1.8 ± 2.4	32	4.26	<.001
2018	2	83.7 ± 3.7	33	77.4 ± 3.6	19	−5.9 ± 2.5	16	9.49	<.0001
2019	1	81.7 ± 3.4	56	79.9 ± 3.8	27	−2.4 ± 2.3	26	5.34	<.0001
2019	2	82.0 ± 4.9	43	76.5 ± 1.7	5	−4.6 ± 1.5	4	5.98	.009
2020	1	81.1 ± 3.5	43	79.1 ± 3.8	15	−1.6 ± 1.7	15	3.72	.002
2020	2	81.2 ± 3.9	34	77.2 ± 2.4	7	−3.8 ± 3.1	7	3.29	.02
2021	1	81.5 ± 3.1	48	79.4 ± 2.8	35	−2.6 ± 2.3	35	6.51	<.0001
2021	2	82.1 ± 4.3	30	77.4 ± 1.7	7	−5.6 ± 2.4	7	6.07	<.001
2022	1	83.1 ± 3.8	26	80.8 ± 3.2	15	−3.2 ± 2.6	15	4.70	<.001
2022	2	83.9 ± 3.8	56	78.6 ± 3.1	32	−5.8 ± 2.7	32	12.00	<.0001

*Note*: All mass data are reported as means ± SD. The first two rows represent all data pooled by year and brood. All *t*‐statistics and *p*‐values are the result of unpaired *t*‐tests with Bonferroni corrections.

For second broods, neither change in body mass (*F*_4,58_ = 1.00, *p* = .42) nor chick‐rearing mass (*F*_6,42_ = 1.80, *p* = .12) varied among years, although mass change between incubation and chick‐rearing stages was significant in all second brood years (Table 1). In contrast, there was significant annual variation in incubation mass (*F*_5,125_ = 7.79, *p* < .0001) with seven significant pairwise differences among six of 7 years (*p* < .05 in all cases), again suggesting that incubation mass was more variable.

Comparing first and second broods within years, incubation mass was significantly greater in second broods during 2018 only (*t*_29_ = −3.07, *p* = .02) while chick‐rearing mass was significantly greater in first broods during 2019 only (*t*_13_ = 3.24, *p* = .04). Birds lost significantly more mass during second broods relative to first broods in both 2018 (*t*_29_ = 5.48, *p* < .0001) and 2022 (*t*_29_ = 3.16, *p* = .02; Table 2). Pooling years, incubation mass was greater in second broods than in first broods (*t*_444_ = −3.42, *p* < .001) while chick‐rearing mass was greater in first broods (*t*_171_ = 3.54, *p* < .001). Mass loss was greater for second broods compared to first broods (*t*_129_ = 8.31, *p* < .0001; Table 2). Pooling years and restricting the dataset to individual females where change in mass data were available for both first and second broods (*n* = 24), mean incubation mass for first and second broods were 82.9 ± 3.7 and 83.3 ± 3.3 g while mean chick‐rearing masses were 80.5 ± 3.3 and 78.0 ± 2.9 g, respectively. Mean mass loss for first and second broods were −2.4 ± 2.2 and −5.1 ± 1.7 g, respectively. Incubation mass did not differ significantly between first and second broods (*t*_23_ = −0.66, *p* = .51). However, chick‐rearing mass was greater during first broods (*t*_23_ = 3.44, *p* = .002) while change in mass was greater during second broods (*t*_23_ = 4.26, *p* < .001).

**TABLE 2:** Interbrood comparisons of incubation mass, chick‐rearing mass, and change in mass within years for adult female European starlings.

Year	Incubation mass				Chick‐rearing mass				Change in mass			
	*n*_1_	*n*_2_	*t*	*p*	*n*_1_	*n*_2_	*t*	*p*	*n*_1_	*n*_2_	*t*	*p*
Pooled	258	229	−3.42	<.001	147	80	3.54	<.001	137	75	8.31	<.0001
2012	31	33	−1.73	.54	19	10	0.66	1.00	14	9	1.96	.45
2018	54	33	−3.07	.02	36	19	1.74	.54	32	16	5.48	<.0001
2019	56	43	−0.31	1.00	27	5	3.24	.04	26	4	2.51	.31
2020	43	34	−0.13	1.00	15	7	1.44	1.00	15	7	1.78	.69
2021	48	30	−0.74	1.00	35	7	2.58	.13	35	7	2.99	.10
2022	26	56	−0.81	1.00	15	32	2.15	.24	15	32	3.16	.02

*Note*: The first row represents all data pooled by year and brood while columns “*n*_1_” and “*n*_2_” represent sample sizes for incubation mass, chick‐rearing mass, and change in mass for first and second broods, respectively. All *t*‐statistics and *p*‐values are the result of unpaired *t*‐tests with Bonferroni corrections.

### 3.2 Individual variation in incubation mass, chick‐rearing mass, and mass loss relative to current reproduction

**FIGURE 1:**
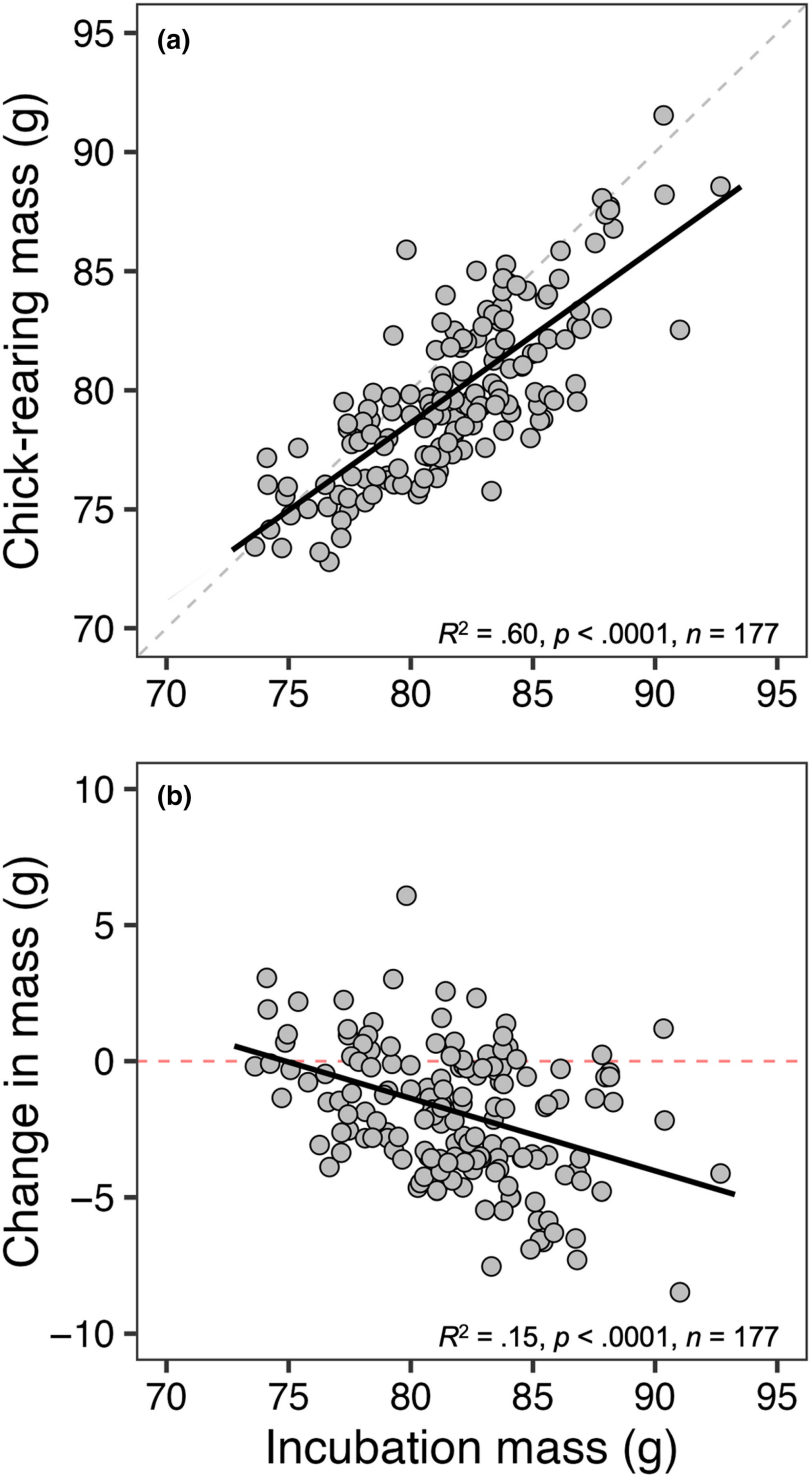
Relationship between incubation mass and (a) chick‐rearing mass and (b) change in mass of adult female European starlings during first broods. Fitted black lines represent lines of best fit on predicted values as a result of linear mixed‐effects models while the gray dashed line in panel A indicates a slope of 1 and the horizontal red dashed line in panel (b) indicates a net zero change in body mass.

**FIGURE 2:**
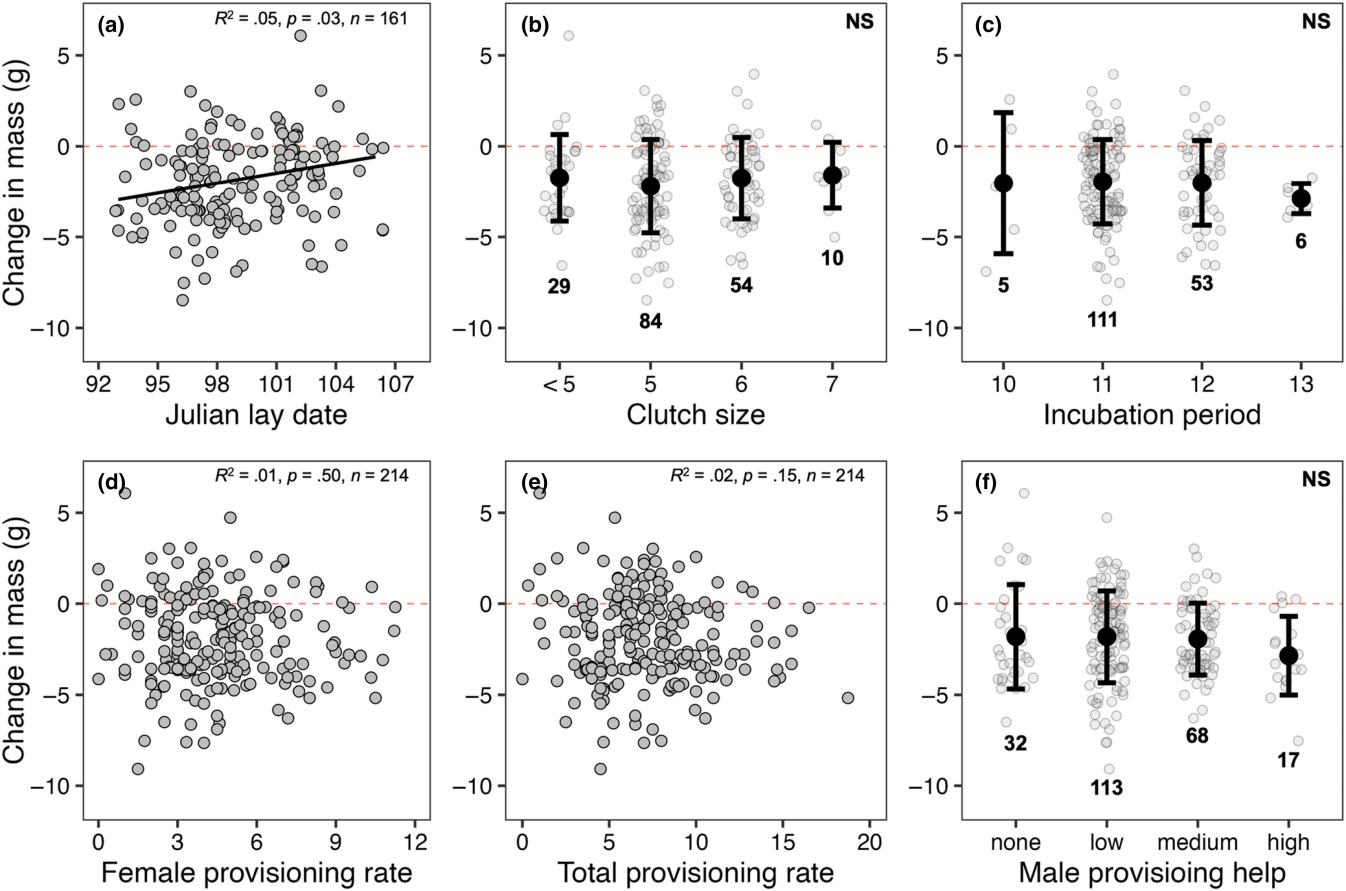
Variation in mass loss of adult female European starlings relative to early season reproductive decisions (a–c) and measures of workload (d–f) during current breeding efforts for first broods. Individuals that laid clutches smaller than four (*n* = 2) were binned into “<5” (b). Provisioning rate was measured as the number of visits per 30 min (d, e) and male help was binned based on male provisioning rates (i.e., >6.00 = high, 3.00–5.99 = medium, 0.01–2.99 = low, 0.00 = none). All provisioning rate data are based on 30‐min video‐recorded provisioning observations and are restricted to days 5–10 of chick‐rearing. The fitted black line in panel (a) represents the line of best fit on predicted values as a result of a linear mixed‐effects model while horizontal red dashed lines indicate a net zero change in body mass. Black circles with vertical bars indicate means ± SD while in‐figure values indicate sample sizes (b, c, f). “NS” = nonsignificant.

**FIGURE 3:**
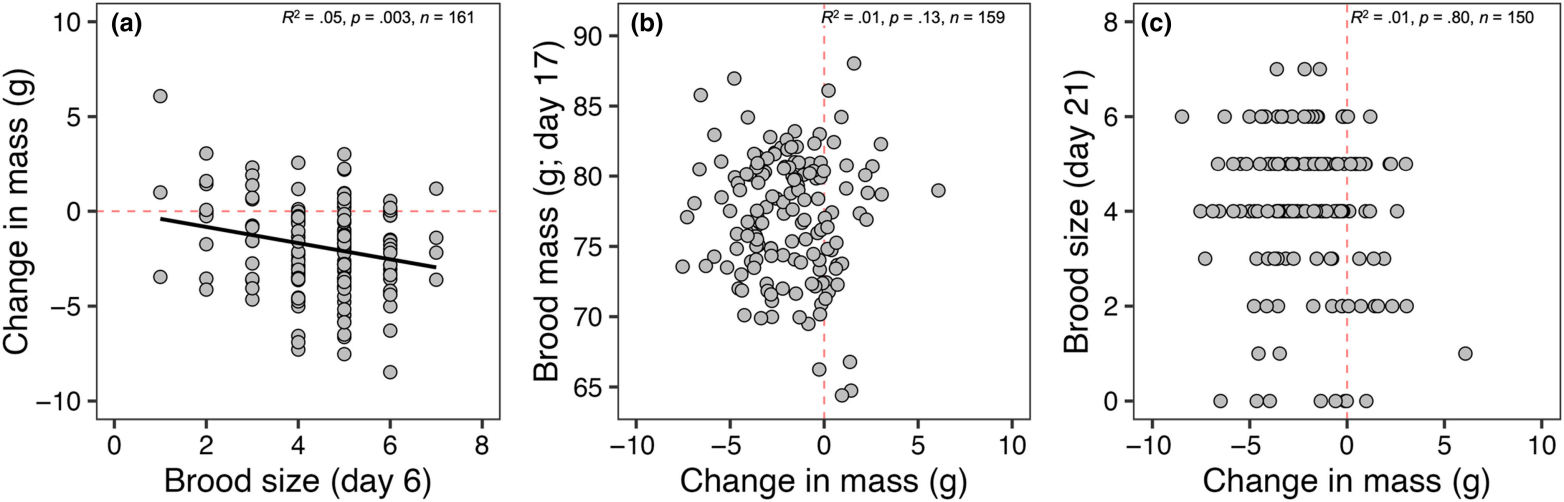
Relationships between change in mass and (a) brood size at day 6, (b) mean brood mass at day 17, and (c) brood size at day 21 for first broods. Brood size at days 6 and 21 represent the number of days post‐hatch (i.e., nestling age) while brood mass represents the mean mass of all nestlings in the brood at day 17 post‐hatch. The fitted black line in panel (a) represents the line of best fit on predicted values as a result of a linear mixed‐effects model while horizontal and vertical red dashed lines indicate a net zero change in body mass.

Chick‐rearing mass had a strong positive correlation with incubation mass (*R* ^2^ = .60, *p* < .0001; Figure 1a) but differed significantly from a slope of 1 (slope = 0.73, Χ^2^(1) = 27.52, *p* < .0001). Change in mass had a weak negative correlation with incubation mass (*R* ^2^ = .17, *p* < .0001; Figure 1b). There was a significant, but weak, relationship between mass loss and Julian lay date (*R* ^2^ = .05, *p* = .03; Figure 2a); however, mass loss was not significantly different based on other early season reproductive decision parameters: clutch size (*F*_3,146_ = 0.59, *p* > .60 in all cases; Figure 2b) and incubation period (*F*_3,131_ = 0.19, *p* > .91 in all cases; Figure 2c). Furthermore, no relationship was observed between mass loss and metrics of workload during current breeding efforts: female provisioning rate (*R* ^2^ = .01, *p* = .50; Figure 2d) and total provisioning rate (*R* ^2^ = .02, *p* = .15; Figure 2e). There was no significant difference in mass loss based on degree of male provisioning contribution (*F*_3,193_ = 1.16, *p* > .48 in all cases; Figure 2f). In testing whether productivity is dependent on mass loss, we found a significant, but weak, correlation between mass loss and brood size at day 6 (*R* ^2^ = .05, *p* = .004; Figure 3a), but no correlation between mass loss and brood mass at day 17 (*R* ^2^ = .01, *p* = .80; Figure 3b), nor mean brood size at day 21 post‐hatch (*R* ^2^ = .01, *p* = .13; Figure 3c).

### 3.3 Individual variation in mass loss relative to future fecundity and survival

**FIGURE 4:**
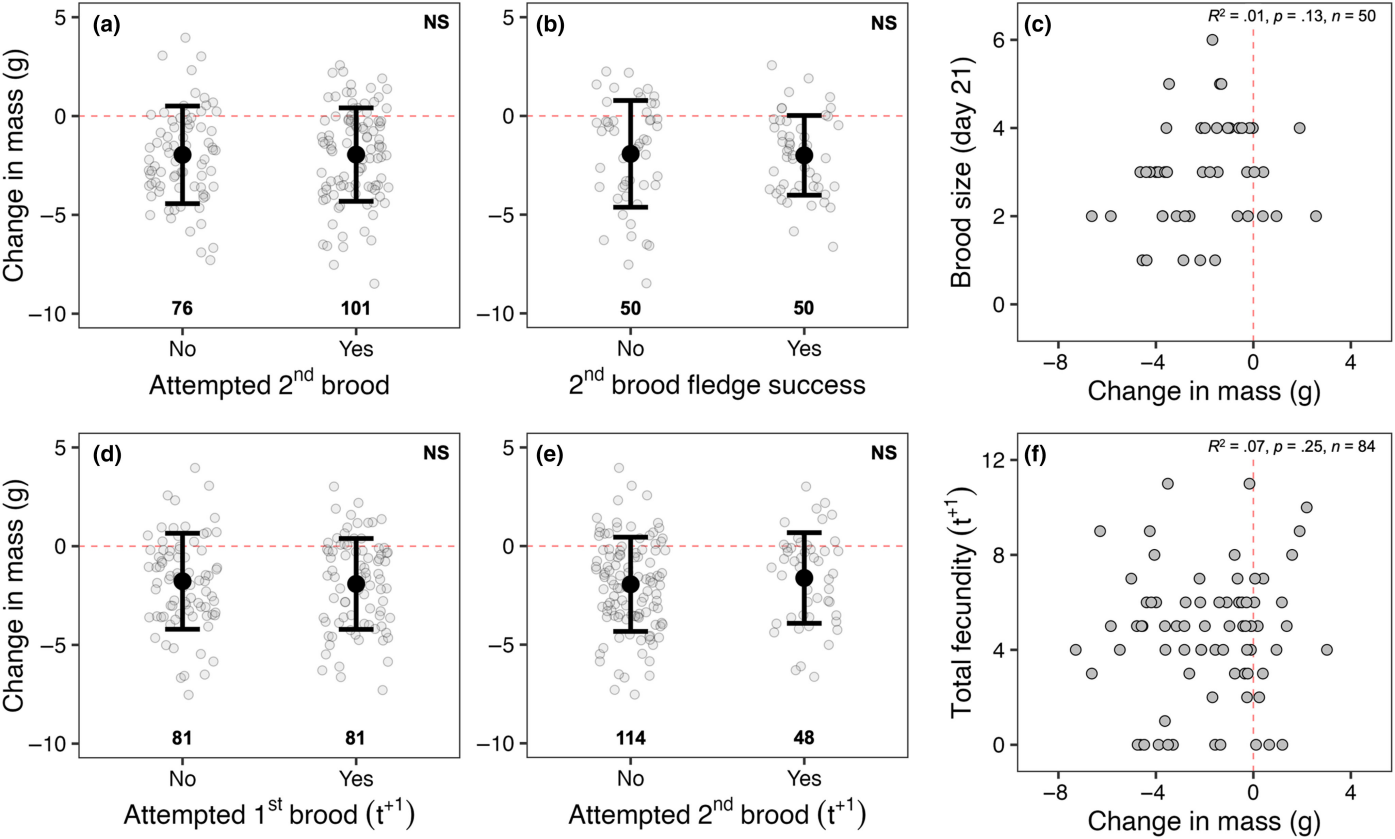
Relationship between change in mass during a first brood attempt and future breeding performance during a second brood attempt in the same year (a–c) and in a subsequent year (i.e., *t* ^+1^; d–f). In panel (f), total fecundity in a subsequent year is defined as the total number of chicks fledged across brood attempts in that year. Horizontal and vertical red dashed lines indicate a net zero change in body mass. In‐figure values represent sample sizes in panels (a, b) and (d, e).

Between 2012 and 2022, 100 of 177 (57%) adult female starlings attempted a second brood in a single breeding season while 50 of these 100 (50%) individuals successfully fledged at least one chick following a second brood attempt. However, change in mass did not differ significantly based on whether an individual attempted a second brood in the same year (*F*_1,131_ = 0.01, *p* = .97; Figure 4a) or whether individuals successfully fledged at least one chick during a second brood attempt (*F*_1,83_ = 0.44, *p* = .51; Figure 4b). Furthermore, we found no relationship between first brood change in mass and the size of second broods at fledge (*R* ^2^ = .01, *p* = .13; Figure 4c) in the same year. In a subsequent year, 81 of 162 (50%) and 48 of 162 (30%) individuals attempted a first and second brood, respectively. Change in mass did not differ significantly based on whether an individual attempted a first brood (*F*_1,102_ = 0.02, *p* = .88; Figure 4d) or a second brood (*F*_1,131_ = 0.76, *p* = .38; Figure 4e) in a subsequent year. Additionally, we found no relationship between first brood change in mass and total fecundity in a subsequent year (*R* ^2^ = .07, *p* = .25; Figure 4f).

### 3.4 Intra‐ and interannual variation and repeatability of mass loss

We found strong positive relationships between first and second brood incubation mass (*R* ^2^ = .52, *p* < .0001) and chick‐rearing mass (*R* ^2^ = .62, *p* < .0001). There was a weak, nonsignificant negative relationship between first and second brood change in mass (*R* ^2^ = .13, *p* = .23). An analysis of intra‐annual repeatability between first and second broods revealed high among‐individual repeatability in incubation mass (*R* = .58 ± .08, *p* < .0001) and chick‐rearing mass (*R* = .79 ± .11, *p* < .0001) but no repeatability in change in mass (*R* = .00 ± .14, *p* = 1.00).

**FIGURE 5:**
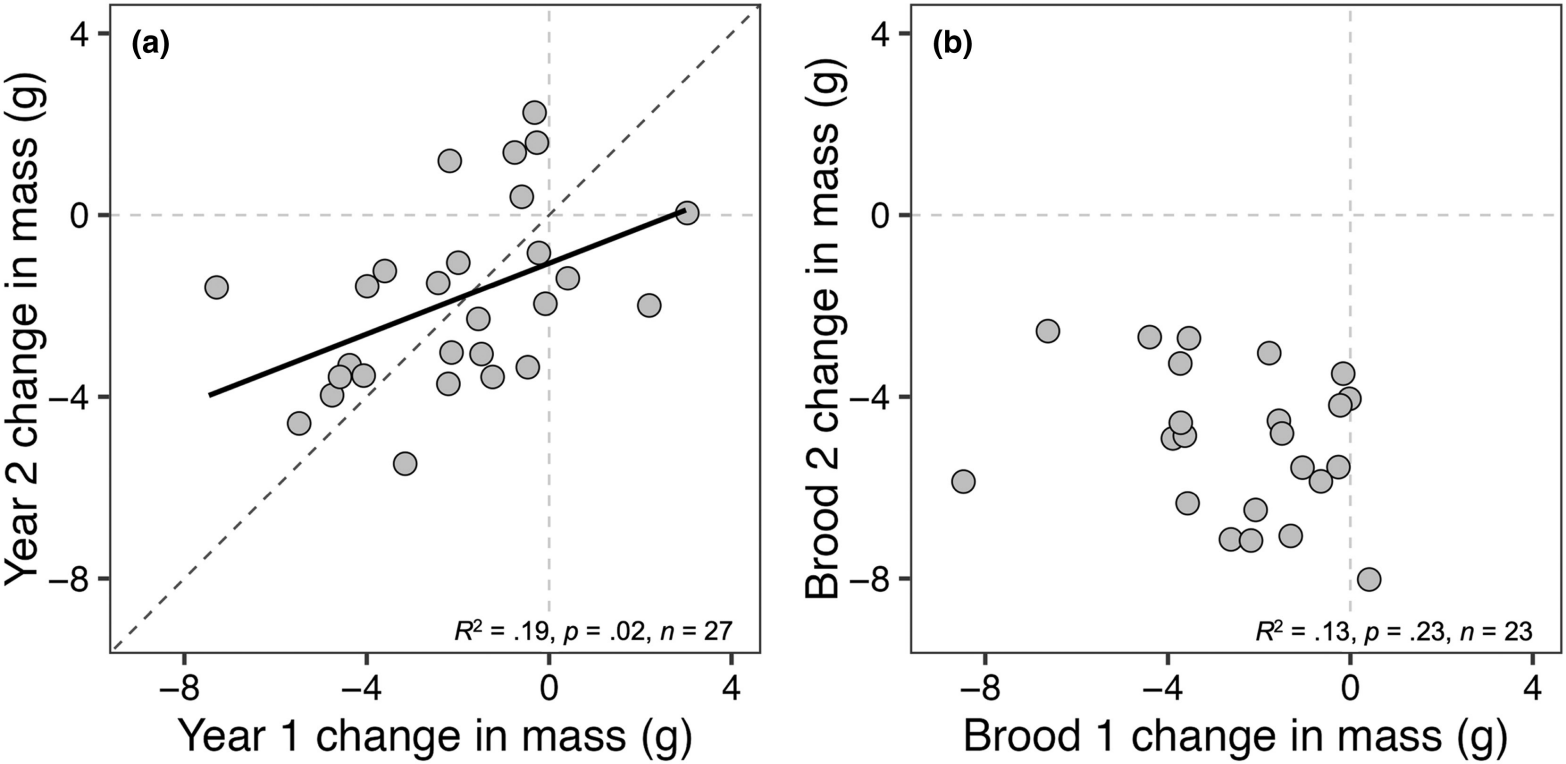
(a) Interannual variation in mass loss during first broods and (b) intra‐annual variation in mass loss across breeding attempts for adult female European starlings. The fitted black line in panel (a) represents the line of best fit on predicted values as a result of a linear mixed‐effects model while, in panels (a, b), the diagonal gray dashed lines indicate a slope of 1 and the horizontal and vertical gray dashed lines indicate a net zero change in body mass.

We found strong positive relationships between year 1 and year 2 incubation mass (*R* ^2^ = .69, *p* < .0001) and chick‐rearing mass (*R* ^2^ = .68, *p* < .0001) and a weak positive relationship between year 1 and year 2 mass loss (*R* ^2^ = .19, *p* = .02; Figure 5a); however, this relationship differed significantly from a slope of 1 (slope = 0.39, Χ^2^(1) = 15.22, *p* < .0001). An analysis of interannual repeatability of first brood data revealed (a) high among‐individual repeatability in incubation mass (*R* = .83 ± .06, *p* < .0001) and chick‐rearing mass (*R* = .85 ± .06, *p* < .0001) and (b) a significant proportion of observed variance attributable to among‐individual differences in mass loss across years (*R* = .50 ± .15, *p* = .002). When incorporating incubation mass into the repeatability analysis as a covariate, the proportion of observed variance attributable to among‐individual differences in mass loss across years increased (*R* = .61 ± .12, *p* < .001). We found no relationship between residual change in mass and change in incubation mass (*R* ^2^ = .03, *p* = .11) nor change in male provisioning rate (visits/30 min; *R* ^2^ = −.01, *p* = .19). Additionally, no other relationships were found between residual change in mass and annual mean‐corrected values for lay date, clutch size, and brood size at fledge, nor measures of workload: male and female provisioning rates, even when correcting for number of chicks (i.e., visits/chick/30 min).

## 4 DISCUSSION

We investigated annual and individual variation in European starling body mass during reproduction, in multiple years, to determine if mass loss represents a phenotypically plastic trait that varies predictably in relation to ecological context and/or individual quality. We found marked annual variation in incubation mass, chick‐rearing mass, and change in mass during first broods but only in incubation mass during second broods. Birds were actually heavier during incubation in second broods but then had greater mass loss by day 10 chick‐rearing relative to first broods (5.4 vs. 2.0 g). We found no support for the idea that mass loss reflects “reproductive stress” in our system: there were no negative relationships between mass loss and either current or future reproduction and survival. In addition, we found moderate repeatability of interannual mass loss during first broods (*R* = .61), though with considerable residual environmental variation, and no repeatability of intra‐annual mass loss between first and second broods when ecological context is very different. Our results are consistent with mass loss being an individually plastic trait (sensu Descamps et al., 2011; McNamara, 1998; McNamara & Houston, 1996), with individuals using mass loss to “level the playing field” and individually optimize reproductive effort and fitness within their specific ecological context and relative to their individual quality for a given breeding attempt.

### 4.1 Annual variation in incubation mass, chick‐rearing mass, and change in mass during reproduction

Despite several studies reporting evidence of birds either adopting a mass loss strategy (Blem & Blem, 2006; Cichoń, 2001; Freed, 1981) or losing mass as a consequence of reproductive stress (Bryant, 1988; De Steven, 1980; Drent & Daan, 1980; Nur, 1984), few studies have considered whether patterns of body mass and mass loss vary predictably in relation to ecological context (i.e., “good” vs. “bad” years). Here, we predicted that, if mass loss represents an adaptive strategy to ameliorate the costs of increased activity during parental care, mass loss would vary across years assuming resources to meet costs of care vary. While there was marked annual variation in incubation mass during both first and second broods, there were fewer differences in chick‐rearing mass and, here, change in mass was largely driven by single year anomalies (e.g., all years relative to 2014; the only year with positive mean mass change between incubation and chick rearing; Table 1). Higher, and more variable, incubation mass could be an artifact of lower quality individuals dropping out of the population prior to our final sampling endpoint (day 10 chick‐rearing), but we found no difference in incubation mass between birds that were and were not resampled during the chick‐rearing stage. Rather, our data support the idea that birds maintain higher mass during incubation as an “insurance” strategy (Potti & Merino, 1995), and higher variance in incubation mass could then be related to females having imperfect knowledge of food availability earlier in the season given selection matches prey abundance to chick rearing later in the season (Williams et al., 2015). This idea also explains higher incubation mass for second broods (i.e., more “insurance,” protecting self‐maintenance) since chick‐rearing conditions are poorer, and overall productivity lower, for second broods in our study system (Cornell & Williams, 2016).

We found systematic mass loss (relative to zero) in seven of 9 years for first broods and all 6 years for second broods (Table 1). This would be predicted especially if females are maintaining higher mass during incubation to cope with overnight fasting and fewer self‐feeding opportunities. Mass loss is theoretically predicted regardless of whether it represents an adaptive strategy or a product of reproductive stress. However, these two hypotheses differ fundamentally in when mass loss is predicted; if adaptive, mass loss should occur at, or just prior to, the onset of peak energetic demands of nestlings, whereas if a product of reproductive stress, mass loss should occur gradually throughout a single nesting cycle, with the greatest loss of mass occurring coincident with peak demands of nestlings. We are unable to disentangle this phenomenon with this study but data on the same population of European starlings using an automated weighing system reveal differential patterns of mass loss between breeding bouts: mass loss occurred abruptly around hatch during first broods and linearly across the entire nesting cycle during second broods (B.L. Hodinka and T.D. Williams, unpublished data). Thus, taken together with our results indicating that second broods lose more mass (−5.4 ± 2.8 g; *n* = 75) relative to first broods (−2.0 ± 2.4 g; *n* = 177; Table 2) and subsequently fledge fewer chicks (1.5 ± 1.7 vs. 3.6 ± 2.0 chicks, respectively), these data may suggest a difference in mass loss strategy linked to reduction in prey availability late in the breeding season.

### 4.2 Individual variation in incubation mass, chick‐rearing mass, and mass loss relative to current reproduction

If mass loss represents an adaptive strategy used to ameliorate the costs of increased activity during parental care through its presumed link to reduced wing loading and increased flight efficiency (Blem, 1976; Norberg, 1981), we should then expect to see consistent positive relationships between mass loss and current reproduction. However, we found little evidence of this here in relation to mass loss (Figures 2a–c and 3). Although birds that lost more mass had larger broods at day 6 post‐hatch, this relationship dissolved by day 21 post‐hatch (Figure 3c). Additionally, while we found a significant relationship between change in mass and lay date where birds that began laying earlier lost more mass, this is confounded by the fact that heavier birds tended to lay earlier (*R* ^2^ = .09, *p* = .001) and lost more mass between stages (Figure 4b). Instead, our data are more consistent with other studies that failed to find robust systematic relationships between mass loss and clutch size (Sandercock, 1997), incubation period (Dobbs et al., 2006), and overall productivity (Gebhardt‐Henrich et al., 1998; Murphy et al., 2000). Furthermore, we predicted that, if mass loss reflects reproductive stress, we should expect to see positive relationships between mass loss and measures of workload (e.g., total provisioning rate) as mass loss is predicted to occur across the entirety of the chick‐rearing phase and be greatest during the peak energetic demands of nestlings. Notably, while some studies have found positive relationships between mass loss and total provisioning rate (Gebhardt‐Henrich et al., 1998; Murphy et al., 2000) and where parents receive no provisioning help (e.g., widowed parents; Sasvari, 1986), our data do not support this (Figure 2d–f). This might be in part because individuals have considerable plasticity in adjusting other aspects of chick provisioning such as load size, prey type, or foraging distance (Wright et al., 1998). Alternatively, differences in individual quality may result in some “higher quality” parents provisioning more efficiently rather than simply working harder (Daunt et al., 2006; Lescroël et al., 2010).

In our population of European starlings, individuals heavier midway through incubation were subsequently heavier at the midpoint of chick rearing relative to their lighter individual counterparts although mass loss was greater for heavier birds (Figure 1). As suggested by Wendeln and Becker (1999), these birds consequently might have been working well below their physiological capacities (4× basal metabolic rate; Drent & Daan, 1980) while birds starting a reproductive cycle lighter at incubation may be living closer to some critical condition threshold with fewer reserves (body mass) to lose by time chick provisioning begins. In this case, we could predict that across years with varying ecological context, many lighter birds should pay a “cost” of living closer to this critical condition threshold. However, we failed to find evidence that birds lighter at incubation suffered greater reproductive failure. Instead—taken together with our repeatability analysis suggesting that heavier birds in a given year tend to be heavier in a subsequent year regardless of environmental condition—our data may suggest that there are inherently “heavy” and “light” body mass phenotypes where heavier birds lose more mass and lighter birds lose less mass. This in turn supports the idea that birds may be individually optimizing reproductive decisions to avoid downstream fitness costs (Aparicio, 1998; Descamps et al., 2011; Pettifor et al., 1988, 2001) and that costs are only to be expected when reproductive adjustments are not optimal. This interpretation is then more consistent with our results and the idea that individuals would be expected to have lower fitness by either losing too much or too little mass given their specific ecological context and/or body condition during that breeding attempt (but see below).

### 4.3 Individual variation in mass loss relative to future fecundity and survival

Numerous experimental studies (reviewed in Williams, 2012) have shown that costs of reproduction, such as those predicted by the reproductive stress hypothesis, are often not apparent during the current breeding attempt but instead are deferred, only becoming apparent through reduced future fecundity and survival (Daan et al., 1996; Nur, 1984; Reid, 1987; Roskaft, 1985) as predicted by life‐history theory (Stearns, 1976; Williams, 1966). To our knowledge, our study is the first to investigate long‐term fitness consequences of female mass loss in terms of future fecundity (in second broods or subsequent years) and survival (local return rate). We predicted that, if mass loss reflected reproductive stress, then there should be consistent negative relationships between mass loss and future fecundity and survival—a “cost of reproduction.” However, we found no support for this prediction (Figure 4). Rather, results from our study match those predicted if birds are individually optimizing reproductive decisions, including mass loss, consequently avoiding downstream fitness costs of reproduction (Aparicio, 1998; Descamps et al., 2011; Pettifor et al., 1988, 2001). As noted by Descamps et al. (2011), assuming a population where individuals do optimize reproductive decisions to their internal and external states, costs of reproduction are only expected when individuals' reproductive adjustments are in any way not optimal (i.e., not properly adjusted to body condition and/or environmental conditions; McNamara & Houston, 1996). Thus, our results support the benefit–cost hypothesis proposed by Hillström (1995) whereby optimal body mass may change with environmental conditions such that higher body mass (and thus more reserves) may be favorable when the environment (or individual quality) is unpredictable and lower body mass favorable when conditions are good (e.g., Ekman & Hake, 1990). In other words, individual females may use mass loss to “level the playing field” and individually optimize current reproductive effort and future fitness.

### 4.4 Intra‐ and interannual variation and repeatability of mass loss

We predicted that, if mass loss was related to reproductive stress, then we should expect low repeatability of mass loss since it is unlikely that individuals would experience the same level of high or low “stress” in subsequent breeding attempts across years. Indeed, we found no repeatability of intra‐annual mass loss between first and second broods, supporting the idea that mass loss is a plastic trait that varies in relation to ecological context; here, it is related to reduced prey availability later in the breeding season with overall much lower breeding success (Cornell & Williams, 2016). In contrast, we found a significant positive relationship between first brood year 1 and year 2 mass loss (Figure 5a) and moderate interannual repeatability of mass loss (*R* = .50), which increased when incorporating incubation mass as a covariate (*R* = .61; Figure 5a). Our results contrast with those of Potti and Merino (1997), who reported low, nonsignificant, repeatability of mass loss in breeding female pied flycatchers (*R* = −.10) when environmental variables were accounted for. However, Potti and Merino (1997) only measured mass loss to the end of the brooding period before females started to feed chicks at comparable rates to males (see Potti & Merino, 1995). Our study suggests, in contrast to the conclusion of Potti and Merino (1997), that there might be significant additive genetic variance for mass loss, though we acknowledge that repeatability estimates do not always set an upper limit to heritability (Dohm, 2002). This is consistent with the idea that, if mass loss truly represents an adaptive strategy, it should be a target for selection and therefore moderate repeatability could suggest some heritability for this phenotypic trait (sensu Wilson, 2018). Even though “individual” explained 61% of variation in mass loss, the remaining 39% of variance attributable to within‐individual differences should then relate to either year‐specific differences in individual quality (e.g., Rands et al., 2006) or differences in environmental conditions experienced by individual females. However, we failed to find any strong relationships between residual change in mass relative to changes in incubation mass, annual mean‐corrected values of lay date, clutch size, and brood size at fledge, nor measures of workload: male and female provisioning rates, even when correcting for the number of chicks. Thus, this residual variation in mass loss remains unexplained but might, for example, reflect other aspects of ecological context, such as resource or food availability or environmental conditions, that we did not measure.

## AUTHOR CONTRIBUTIONS

**Brett L. Hodinka:** Conceptualization (equal); data curation (lead); formal analysis (lead); methodology (lead); visualization (lead); writing – original draft (lead); writing – review and editing (equal). **Tony D. Williams:** Conceptualization (equal); funding acquisition (lead); supervision (lead); writing – review and editing (equal).

## CONFLICT OF INTEREST STATEMENT

No conflicts of interest, financial or otherwise, are declared by the authors.

## Data Availability

The data that support the findings of this study are openly available in the Dryad data repository at http://doi.org/10.5061/dryad.4j0zpc8jj. “Private for Peer Review” link: https://datadryad.org/stash/share/FqfbDU3FDEABfUirGWSVYQd6n77YzeeCCqqrD8F‐VrA.
